# Mutational Analysis of the GXXXG/A Motifs in the Human Na^+^/Taurocholate Co-Transporting Polypeptide NTCP on Its Bile Acid Transport Function and Hepatitis B/D Virus Receptor Function

**DOI:** 10.3389/fmolb.2021.699443

**Published:** 2021-06-22

**Authors:** Massimo Palatini, Simon Franz Müller, Kira Alessandra Alicia Theresa Lowjaga, Saskia Noppes, Jörg Alber, Felix Lehmann, Nora Goldmann, Dieter Glebe, Joachim Geyer

**Affiliations:** ^1^ Institute of Pharmacology and Toxicology, Faculty of Veterinary Medicine, Justus Liebig University Giessen, Giessen, Germany; ^2^ Institute of Medical Virology, National Reference Center for Hepatitis B and D Viruses, Justus Liebig University Giessen, Giessen, Germany

**Keywords:** Na^+^/taurocholate co-transporting polypeptide, bile acid transport, dimerization, sorting, protein-protein interaction, hepatitis B virus, hepatitis D virus

## Abstract

Homodimerization is essential for plasma membrane sorting of the liver bile acid transporter NTCP and its function as Hepatitis B/D Virus (HBV/HDV) receptor. However, the protein domains involved in NTCP dimerization are unknown. NTCP bears two potential GXXXG/A dimerization motifs in its transmembrane domains (TMDs) 2 and 7. The present study aimed to analyze the role of these GXXXG/A motifs for the sorting, function, and dimerization of NTCP. The NTCP mutants G_60_LXXXA_64_L (TMD2), G_233_LXXXG_237_L (TMD7) and a double mutant were generated and analyzed for their interaction with wild-type NTCP using a membrane-based yeast-two hybrid system (MYTH) and co-immunoprecipitation (co-IP). In the MYTH system, the TMD2 and TMD7 mutants showed significantly lower interaction with the wild-type NTCP. In transfected HEK293 cells, membrane expression and bile acid transport activity were slightly reduced for the TMD2 mutant but were completely abolished for the TMD7 and the TMD2/7 mutants, while co-IP experiments still showed intact protein-protein interactions. Susceptibility for *in vitro* HBV infection in transfected HepG2 cells was reduced to 50% for the TMD2 mutant, while the TMD7 mutant was not susceptible for HBV infection at all. We conclude that the GXXXG/A motifs in TMD2 and even more pronounced in TMD7 are important for proper folding and sorting of NTCP, and so indirectly affect glycosylation, homodimerization, and bile acid transport of NTCP, as well as its HBV/HDV receptor function.

## Introduction

The Na^+^/taurocholate co-transporting polypeptide (NTCP, gene symbol *SLC10A1*) is the first of seven members of the solute carrier family SLC10 (Geyer et al., 2006) and plays, together with the apical sodium bile acid transporter (ASBT, gene symbol *SLC10A2*), a crucial role for the maintenance of the enterohepatic circulation of bile acids (Döring et al., 2012). While NTCP is dominantly expressed in hepatocytes and here is responsible for the re-uptake of bile acids from the portal blood (Stieger et al., 1994), ASBT, with its highest expression level in the apical brush border membrane of enterocytes of the terminal ileum, absorbs bile acids from the intestinal lumen for their return to the liver (Shneider, 1995). The identification of NTCP as the high-affinity binding and entry-receptor for the human Hepatitis B (HBV) and Hepatitis D (HDV) Viruses in 2012 made this carrier an attractive novel drug target for HBV/HDV entry inhibition (Yan et al., 2012; König et al., 2014; Kirstgen et al., 2020).

NTCP forms homodimers, in which the individual subunits are functionally active in transporting bile acids in a sodium-dependent manner (Bijsmans et al., 2012; Noppes et al., 2019). NTCP homodimerization occurs early in the secretory pathway and persists after its sorting to the plasma membrane (Bijsmans et al., 2012). In addition, NTCP dimerization seems to be essential for the entry of HBV/HDV virus particles into hepatocytes (Fukano et al., 2018). Interestingly, after co-expression of NTCP with the NTCP homolog SLC10A4, which has a vesicular expression pattern, wild-type NTCP is trapped in intracellular compartments and so plasma membrane expression and bile acid transport function of NTCP are hampered (Bijsmans et al., 2012; Noppes et al., 2019). These data clearly point to functional homo- and heterodimerization of NTCP, but the protein domains responsible for this interaction have not been identified so far. Two GXXXG/A sequence motifs are present in transmembrane domains (TMDs) 2 and 7 of NTCP. Such motifs are well-known to be involved in protein-protein interactions of transmembrane proteins (Teese and Langosch, 2015). In the present study, we hypothesize that this sequence motif is involved in the dimerization of NTCP. In detail, this GXXXG/A motif, or more general (small)XXX(small) motif, is typically flanked by the small amino acid residues glycine, alanine or serine (Dawson et al., 2002). It was first described in the blood-based glycophorin A (GPA), a transmembrane sialoglycoprotein in erythrocytes, as a key motif for homodimerization (Lemmon et al., 1992). Since then, this motif has been investigated in numerous other transmembrane proteins (Teese and Langosch, 2015). In addition, random sequence library screening of selected oligomerizing transmembrane domains by the TOXCAT *in vivo* selection system revealed dominant occurrence of the GXXXG motif in transmembrane helix-helix associations (Russ and Engelman, 2000), demonstrating the importance of this motif for transmembrane interactions. According to structure-based studies, the mode of action of this motif might be the enhancement of van der Waals forces and/or hydrogen bonds due to the close proximity of the small amino acid residues (glycine, alanine, or serine) of this motif in the three-dimensional structure of an alpha helix (MacKenzie et al., 1997; Anderson et al., 2017). In addition to intermolecular protein-protein interactions, this (small)XXX(small) motif is also relevant for intramolecular interactions, which are important for proper protein folding and sorting (Eilers et al., 2000). Therefore, the present study aimed to analyze the role of the two GXXXG/A motifs of human NTCP for its transporter and virus receptor functions, sorting and dimerization. Mutation of these motifs in NTCP had significant impact on protein folding and sorting, and so indirectly affected homodimerization and bile acid transport of NTCP, as well as its HBV/HDV receptor function.

## Materials and Methods

### Chemicals

All of the chemicals, unless otherwise stated, were bought from Sigma-Aldrich (St. Louis, MO, United States). Radio-labelled [^3^H]taurocholic acid ([^3^H]TC, 10 Ci/mmol) was purchased from PerkinElmer Life Sciences (Waltham, MA, United States).

### Cell Lines and Transient Transfections

GripTite HEK293 MSR cells (Thermo Fisher Scientific), further referred to as HEK293 cells, were cultured in DMEM (Gibco, Carlsbad, United States) supplemented with 10% fetal calf serum (Pan-Biotech, Aidenbach, Germany), l-Glutamine (4 mM, anprotec, Bruckburg, Germany), penicillin (100 U/ml, anprotec), and streptomycin (100 μg/ml, anprotec) in a 5% CO_2_ atmosphere at 37°C. Human hepatoma HepG2 Tet-On cells (BD Clontech, Heidelberg, Germany), further referred to as HepG2 cells, were maintained under the same conditions in DMEM with all supplements listed above. HEK293 cells were transiently transfected with Lipofectamine 2000 (Thermo Fisher Scientific) for co-localization as well as co-immunoprecipitation (co-IP) experiments following the manufacturer’s protocol. HepG2 cells were transiently transfected using X-tremeGENE 9 (Roche Diagnostics, Basel, Germany) and used for HBV infection experiments.

### Yeast-Two-Hybrid Membrane Protein System

The yeast-two-hybrid membrane protein system (MYTH) enables the identification of interactions between membranous proteins by utilizing the split-ubiquitin method (Stagljar et al., 1998). For this method the proteins of interest have to be fused with either the C-terminal part (C_Ub_/bait construct) or the N-terminal part (N_ub_/prey construct) of ubiquitin, which allows the functional restoration of split-ubiquitin by interaction of the proteins of interest with each other (Johnsson and Varshavsky, 1994). Ubiquitin-specific proteases recognize and cleave the newly formed split-ubiquitin resulting in the separation of the transcriptional factor LexA-VP16 from the C_Ub_ construct, which subsequently activates certain reporter genes (Stagljar and Fields, 2002). All vectors and the reporter strain NMY51 (MATa his3∆200 trp1-901 leu2-3,112 ade2 LYS2::(lexAop)4-HIS3 ura3::(lexAop)8-lacZ ade2::(lexAop)8-ADE2 GAL4) were purchased from DUALsystems Biotech AG (Schlieren, Switzerland). The bait vectors contain a kanamycin resistance gene for selection in chemical competent *E. coli* and a leucine synthesis gene for selection of the NMY51 yeast strain. In contrast, the prey vectors have an ampicillin resistance gene for selection in *E. coli* and a tryptophan synthesis gene for selection in yeast. For co-transformation control, the NMY51 yeast cells were grown on synthetically defined (SD) medium lacking leucine and tryptophan (SD-LW). For the protein interaction assays, the SD medium was deficient in adenine, histidine, leucine, and tryptophan (SD-AHLW). Cloning of the open reading frame of NTCP into the bait vector pBT3-STE and the prey vector pPR3-STE was reported before (Noppes et al., 2019).

### Transformation of Bait and Prey Constructs Into NMY51

The NMY51 yeast strain was grown on YPAD plates containing 1% yeast extract (Roth, Karlsruhe, Germany), 2% tryptone/peptone ex casein (Roth), 2% glucose monohydrate (Roth), 2% agar-agar Kobe I (Roth), and 0.004% adenine sulfate. For transformation, several yeast colonies were inoculated in 50 ml YPAD medium, composed of the same contents as the YPAD plates, but without agar-agar, and grown overnight at 30°C under shaking at 180 rpm. The culture with an OD_600_ of approximately 0.8 was then pelleted and re-suspended in 2.5 ml water. Co-transformations were carried out using 1.5 µg of each plasmid, 300 µl PEG/LiOAc master mix (composed of 2.4 ml 50% PEG 4000 (Roth), 360 µl 1 M lithium acetate (Roth), and 250 µl single stranded carrier DNA (ssDNA) for 10 reactions) and 100 µl of re-suspended yeast cells. Each reaction was incubated at 42°C for 45 min prior to pelleting and resuspending in 100 µl 0.9% NaCl. For single transformations, the resuspended yeast cells were plated on SD-L (bait constructs) or SD-W (prey constructs) plates, containing 0.7% yeast nitrogen base without amino acids (Roth), 0.1% yeast synthetic drop-out medium supplemented with the appropriate amino acids (Roth), 2% glucose monohydrate, and 2% agar-agar Kobe I, respectively. For co-transformations of bait and prey constructs, 4 µl of the yeast/NaCl-suspension were dropped onto SD-LW plates for transformation control and another 4 µl on SD-AHLW plates for interaction analysis. The residual suspension was completely plated on SD-AHLW plates for colony quantification. All plates were incubated at 30°C for 5 days.

### Test for Non-specific Interactions of the Prey Constructs

To test the prey constructs for non-specific interactions, all prey constructs were co-transformed with the control bait construct pTSU2-APP, as described in the DUALmembrane pairwise interaction kit by DUALsystems Biotech AG. Expression of the pTSU2-APP construct leads to the expression of an apolipoprotein-precursor-C_Ub_-LexA-VP16 fusion protein. After plating the transformed yeast cells on SD-AHLW, the plates were incubated for 5 days at 30°C. In this assay, an absence of colonies indicates lack of non-specific interactions.

### Expression Verification of the Prey Constructs

Protein expression of the mutated prey constructs was verified by western blot analysis. The NMY51 yeast strain was transformed with the prey constructs and grown overnight at 30°C under shaking in 10 ml SD-W medium. Membrane proteins were extracted as described (Karginov and Agaphonov, 2016). The samples were loaded onto a 12% SDS polyacrylamide gel and after separation transferred to Hybond electrochemiluminescence (ECL) nitrocellulose membrane (GE Healthcare, Little Chalfont, Buckinghamshire, United Kingdom). Blocking of the membranes was done with 5% low-fat powdered milk (Roth) in TBS-T [137 mM NaCl, 10 mM Tris (Roth), pH 8.0, 0.05% Tween-20 (Roth)] for 60 min prior to an overnight exposure with the primary antibody at 4°C in blocking solution. Detection of prey fusion proteins was performed using a mouse monoclonal anti-HA antibody (HA-Tag Monoclonal Antibody 5B1D10, 1:500, Thermo Fisher Scientific, Waltham, MA, United States, Cat #32-6700, Lot #QC215112). After three washing steps in TBS-T the membrane was incubated with a horseradish peroxidase (HRP)-labelled rabbit polyclonal anti-mouse antibody (anti-mouse IgG, 1:3000, Sigma-Aldrich, St. Louis, Missouri, United States, Cat #A9044, Lot #018M4899V). The western blot was visualized using the Intas ChemoStar and the Intas ChemoStar Imager software.

### Site-Directed Mutagenesis

The mutated NTCP constructs were generated by site-directed mutagenesis as reported before (Bennien et al., 2018), using previously reported templates (König et al., 2014; Noppes et al., 2019) depending on the individual experiment, i.e., NTCP-prey construct, NTCP-bait construct, NTCP-GFP, NTCP-FLAG, and NTCP-V5-His, respectively. The GXXXG/A motifs in TMD2 and TMD7 were mutated into LXXXL using oligonucleotide primers synthesized from Metabion International AG (Planegg, Germany) (see Table 1). All mutated sequences were sequence-verified by Sanger sequencing (Microsynth AG, Balgach, Switzerland).

**TABLE 1:** Oligonucleotide primers used for site-directed mutagenesis of NTCP.

Primer	Sequence (5′→3′)
NTCP_G60L_A64L_forward	gaa​gcc​taa​a**ctg**ctg​gcc​atc**ctc**ctg​gtg​gca​cag​tat​g
NTCP_G60L_A64L_reverse	cat​act​gtg​cca​cca​g**gag**gat​ggc​cag**cag**ttt​agg​ctt​c
NTCP_G233L_G237L_forward	ccc​tga​tgc​ctt​tta​tt**ctc**ttt​ctg​ctg**ctt**tat​gtt​ctc​tct​g
NTCP_G233L_G237L_reverse	cag​aga​gaa​cat​a**aag**cag​cag​aaa**gag**aat​aaa​agg​cat​cag​gg

Bold letters in the respective primer sequences indicate the codons changed for leucine.

### Colocalization in Transfected HEK293 Cells

For colocalization studies, GFP- and mScarlet-tagged NTCP constructs were transiently transfected into HEK293 cells as described (Müller et al., 2018). Cells were seeded into µ-Slide chambered coverslips (Ibidi, Martiensried, Germany) and transfected with 0.25 µg of each appropriate construct pDNA using Lipofectamine 2000 (Thermo Fisher Scientific). Finally, 50 µl of the Lipofectamine-pDNA-complex were added to the cells before incubation was started at 37°C for 2 days. Staining of the nuclei was performed with Hoechst33342 (1 µg/ml, Thermo Fisher Scientific). Fluorescence microscopy was performed on a Leica DMI6000 B fluorescence microscope (Leica, Wetzlar, Germany). All images were taken and analyzed with the Leica software LAS X.

### Co-Immunoprecipitation and Western Blotting

HEK293 cells were transiently transfected with the respective mutants of the NTCP-V5-His and/or NTCP-FLAG constructs, being G_60_LXXXA_64_L (TMD2), G_233_LXXXG_237_L (TMD7), and the double mutant G_60_LXXXA_64_L/G_233_LXXXG_237_L. Six well plates were seeded with HEK293 cells and transfected with 1.2 µg of each pDNA of the respective constructs by Lipofectamine 2000 and incubated for 48 h at standard culture conditions. All following steps were performed at 4°C, if not otherwise indicated. Cells were washed with phosphate-buffered saline (PBS, containing 137 mM NaCl, 2.7 mM KCl (Roth), 1.5 mM KH_2_PO_4_ (Roth), 7.3 mM Na_2_HPO_4_ (Roth), pH 7.4, 37°C) prior to be harvested in 400 µl co-IP lysis buffer consisting of 20 mM Tris-HCl (Roth), 135 mM NaCl (Roth), 10% glycerol (Roth) and 1% Nonidet P40 (BioChemica). After centrifugation at 10,000 *g* for 10 min, the amount of protein in each sample was determined using the BCA Protein Assay Kit (Novagen, St. Louis, United States). Then, the samples were set to 500 µg of total protein. The samples were mixed with 30 µl of Pierce Anti-Flag Magnetic Agarose (Invitrogen, Carlsbad, CA, United States) and incubated overnight in a rotation stand. The agarose was washed three times using co-IP lysis buffer and then heated at 95°C for 10 min with Laemmli sample buffer containing 2% SDS (Roth), 10% glycerol (Roth), 0.002% bromophenol blue (Merck), 62.5 mM Tris-HCl (Roth) and 5% 2-mercaptoethanol (Roth). After cooling to room temperature, samples were loaded onto a 12% SDS polyacrylamide gel. Western blotting was performed as described above. The fusion proteins were detected using an anti-V5 rabbit polyclonal antibody (1:3000, Sigma-Aldrich, Cat #V8137, Lot #21160752) or an anti-FLAG rabbit polyclonal antibody (1:2000, Sigma-Aldrich, Cat #F7425, Lot #078M4886V). Glyceraldehyde 3-phosphate dehydrogenase (GAPDH) served as loading control and was detected using an anti-GAPDH polyclonal antibody (1:2000, Sigma-Aldrich, Cat #SAB2500450, Lot #6377C3). As secondary antibodies, a HRP-labelled goat polyclonal anti-rabbit antibody (Invitrogen, Cat #31460, Lot #UK293475) and a HRP-labelled rabbit polyclonal anti-goat antibody (Invitrogen, Cat #81-1620, Lot #VH308190) were used. The western blots were visualized using the Intas ChemoStar and the Intas ChemoStar Imager software. For quantification of the band intensities, the raw images of 10-min exposure time were used and the whole lane intensities were measured using ImageJ (Schneider et al., 2012). The quotient of the intensities between the co-IP (anti-V5) and the lysate (anti-FLAG) bands was calculated and the quotient of wild-type NTCP was set to 100%.

### Deglycosylation of Na^+^/Taurocholate Co-Transporting Polypeptide

HEK293 cells were mono-transfected with the mutated NTCP-FLAG constructs and lysed at 48 h post transfection as described above. After determination of the protein concentration, 10X Glycoprotein Denaturing Buffer containing 5% SDS and 0.4 M DTT (New England Biolabs, Massachusetts, United States, Cat #P0704S, Lot #0361002) were added to 50 µg of total protein. Samples were heated at 95°C for 10 min. Afterwards, each sample was mixed with a mastermix containing 10X G7 Reaction Buffer (composed of 0.5 M sodium phosphate, pH 7.5), 10% NP-40, and PNGase F (New England Biolabs, Massachusetts, United States, Cat #P0704S, Lot #0361002) followed by an incubation step at 37°C for 1 h. Glycosylated and deglycosylated NTCPs were visualized by western blotting using the Intas ChemoStar and the Intas ChemoStar Imager software. For quantification of the band intensities, the intensities of the area of interest between 50 and 60 kDa in the glycosylated NTCP samples were determined using ImageJ. The intensity of wild-type NTCP was set to 100%.

### Binding Experiments With the Hepatitis B Virus/Hepatitis D Virus-Derived preS1_2-48_ Peptide

HBV and HDV attach to NTCP via their myristoylated preS1-lipopeptide comprising the N-terminal amino acids 2-48 of the large HBV surface protein, briefly called preS1 (Glebe et al., 2005; Glebe and Bremer, 2013). The preS1-mediated HBV/HDV attachment to NTCP triggers virus entry into hepatocytes (Yan et al., 2015; Li and Urban, 2016). Therefore, preS1 peptide binding to NTCP not only indicates plasma membrane expression of NTCP, but also is used as a surrogate susceptibility parameter for *in vitro* HBV/HDV infection (Müller et al., 2018). Here, a preS1 peptide C-terminally labeled with the fluorescent dye AlexaFluor568 (further referred to as preS1-AF568, Biosynthesis, Lewisville, Texas, United States) was used for binding experiments on NTCP-transfected HEK293 cells as described before (König et al., 2014) in order to indicate plasma membrane expression of NTCP. HEK293 cells, expressing the green fluorescent wild-type or mutant NTCP-GFP fusion protein (see above) were incubated with 50 nM preS1-AF568 peptide in DMEM for 20 min at 37°C. After intensive washing with PBS, GFP-derived green fluorescence as well as AF568-derived red fluorescence were analyzed on a Leica DMI6000 B fluorescent microscope.

### Hepatitis B Virus Infection Experiments

HepG2 cells were inoculated with 50,000 genome equivalents of HBV particles per cell for 16 h. HBV was produced *in vitro* as reported before (König et al., 2014). For infection experiments, hepatocyte growth medium (HGM) was used, consisting of William’s E Medium (Thermo Fisher Scientific) supplemented with 2% bovine serum albumin (Roth), 2 mM l-Glutamine (Thermo Fisher Scientific), 100 μg/ml gentamicin (Thermo Fisher Scientific), 10 nM dexamethasone (Sigma-Aldrich), 1 mM sodium pyruvate (Thermo Fisher Scientific), and 1X Insulin-Transferrin-Selen (Thermo Fisher Scientific). During the 16 h infection period, 2% DMSO (Merck, Darmstadt, Germany), 4% polyethylene glycol 8,000 (PEG; Sigma-Aldrich), a mix of antibiotics and antimycotics as well as 100 ng/ml human epidermal growth factor (EGF; Peprotech, Cranbury, New Jersey, United States) were added as reported (König et al., 2014; Müller et al., 2018). Cells were maintained with HGM lacking PEG and EGF. Medium was changed every three days until fixation at day 12 post infection with 3% paraformaldehyde (Sigma-Aldrich) in PBS for 30 min at room temperature (RT). 0.2% Triton X 100 (Roth) in PBS for 30 min at RT was utilized to permeabilize cells, followed by blocking unspecific epitopes with 5% bovine serum albumin (Roth) in PBS for 30 min at RT. For detection of HBV core (HBc) protein as an indicator of HBV infection, cells were incubated for 1 h at 37°C with a polyclonal guinea pig-HBcAg antiserum (1:1000 dilution) and thereafter with anti-guinea pig IgG AF568 (1:800 dilution, Thermo Fisher Scientific Cat# A-11075, Lot# 1885925) for 1 h at 37°C. Nuclei were stained with Hoechst33342 (1 µg/ml, Thermo Fisher Scientific).

### Transport Function of the Na^+^/Taurocholate Co-Transporting Polypeptide GXXXG/A Mutants

Functional characterization of the mono- and co-expressed NTCP wild-type and mutant constructs was performed after transient transfection into HEK293 cells as described previously (Müller et al., 2018). HEK293 cells were cultured as described above and were plated into 24-well plates (Sarstedt, Nümbrecht, Germany) with 3 × 10^5^ cells per well for the transport experiments. Cells were transfected with the indicated constructs with the identical absolute amount of 0.5 μg pDNA per well by Lipofectamine 2000. After 48 h of incubation, cells were washed three times with PBS. Then, cells were pre-incubated with transport buffer (142.9 mM NaCl, 4.7 mM KCl, 1.2 mM MgSO_4_ (Roth), 1.2 mM KH_2_PO_4_, 1.8 mM CaCl_2_ (Roth), and 20 mM HEPES (Roth), pH 7.4, 37°C) for 5 min. For uptake experiments, cells were incubated with 300 µl transport buffer containing 10 µM taurocholic acid (TC) spiked with [^3^H]TC for 10 min at 37°C. Uptake studies were terminated by removing the transport buffer followed by five washing steps in PBS at 4°C. Afterwards, cells were lysed in 1 N NaOH (Roth) with 0.1% SDS and the cell-associated radioactivity of the lysate was determined by liquid scintillation counting. Additionally, protein content per well was determined for data normalization as described (Geyer et al., 2007).

### Homology Model of Human Na^+^/Taurocholate Co-Transporting Polypeptide

A 3D homology model of the human NTCP protein (GenBank accession No. NP_003040) was calculated with the SWISS-MODEL tool (https://swissmodel.expasy.org/) based on the crystal structure of the bacterial homolog ASBT from *Neisseria meningitidis* (PDB: 3zuy). Within this model the N-terminus of NTCP is oriented to the outside and the C-terminus to the intracellular site, according to experimental data (König et al., 2014). Amino acids 1–26 of the NTCP N-terminus as well as amino acids 309-349 of the C-terminus could not be included in the models, meaning that the NTCP homology model only covers amino acids 27-308, from TMD1 to TMD9.

## Results

### Na^+^/Taurocholate Co-Transporting Polypeptide Homodimerization in the Membrane-Based Yeast-Two Hybrid System

**FIGURE 1:**
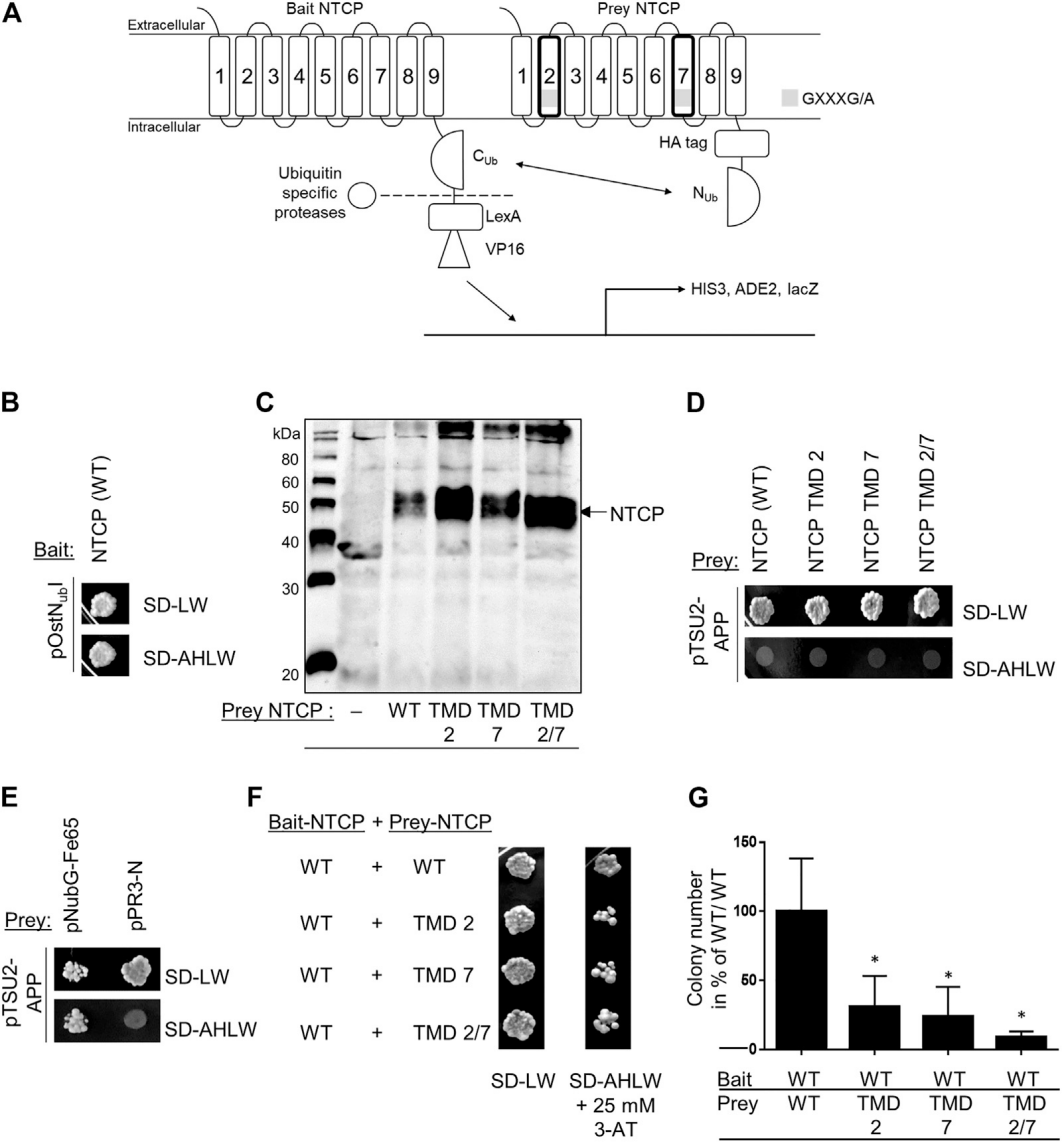
Functional NTCP homodimerization in the MYTH system. **(A)** The MYTH system is based on the fusion of the C-terminal part of ubiquitin with the N-terminal part leading to the separation of the transcriptional factors LexA-VP16 by ubiquitin-specific proteases and activation of genes responsible for the synthesis of histidine and adenine. **(B)** Yeast cells were co-transformed with the wild-type (WT) NTCP-C_ub_-LexA-VP16 bait and the pOstN_ub_I control prey constructs. Growth was analyzed on SD-LW plates (lacking leucine and tryptophan) and on SD-AHLW plates (additionally lacking adenine and histidine). **(C)** Western blot analysis of the wild-type and mutant prey NTCP-HA-N_ub_G fusion proteins with an anti-HA antibody. **(D)** Co-transformation of the wild-type and mutant NTCP prey constructs with the pTSU2-APP control construct and dripping on SD-LW and SD-AHLW plates. **(E)** Interaction control after co-transformation of the pTSU2-APP control construct with the pN_ub_G-Fe65 prey construct (positive control) or with the pPR3-N empty vector (negative control) and dripping on SD-LW and SD-AHLW plates. **(F)** Co-transformation of the NTCP wild-type bait construct with the indicated wild-type or mutant NTCP prey constructs and dripping on SD-LW (co-transformation control) and SD-AHLW (protein-protein interaction assay) plates. The SD-AHLW plates additionally contained 25 mM 3-AT to suppress unspecific interactions. **(G)** The bait-prey co-transformations shown in **(F)** were completely plated on 10 cm SD-AHLW plates containing 25 mM 3-AT and the total number of colonies was counted using OpenCFU (Geissmann, 2013). Data represent means ± SD of four independent experiments. *Significantly different from control (NTCP(WT)-bait/NTCP(WT)-prey) with *p* < 0.01 (one-way ANOVA with Dunnett‘s multiple comparison test).

In order to analyze the role of the G_60_XXXA_64_ and G_233_XXXG_237_ sequence motifs for the dimerization of NTCP, the MYTH system was used to study dynamic protein-protein interactions of membrane proteins. Apart from the C-terminally C_ub_-LexA-VP16-tagged bait and C-terminally HA-N_ub_G-tagged prey wild-type NTCP constructs, the NTCP mutant prey constructs G_60_LXXXA_64_L (TMD2), G_233_LXXXG_237_L (TMD7), and G_60_LXXXA_64_L/G_233_LXXXG_237_L (TMD2/7) were generated by site-directed mutagenesis. Successful co-transformation of the yeasts with the wild-type bait and the mutant prey NTCP constructs was verified in all experiments by plating on SD-LW plates, lacking the amino acids leucine and tryptophan (Figure 1A). Expression of the NTCP-C_ub_-LexA-VP16 bait construct was confirmed by co-transformation with the pOst-N_ub_I control prey vector. In this assay, N_ub_I expression together with NTCP-C_ub_ reconstitutes split-ubiquitin, and so enables yeast growth on the SD-LW (co-transformation control) and SD-AHLW plates (control interaction assay) as shown in Figure 1B. This control assay indirectly confirms expression of the NTCP-C_ub_-LexA-VP16 fusion protein in the yeast cells. In addition, protein expression from the prey NTCP constructs was confirmed by western blot analysis (Figure 1C). Yeast cells were transformed with the respective wild-type or mutant prey NTCP constructs and the extracted proteins were subjected to sodium dodecyl sulfate-polyacrylamide gel electrophoresis (SDS-PAGE). The C-terminally HA-tagged proteins were detected by an anti-HA antibody. Next, non-specific interactions of the NTCP mutant prey constructs were analyzed. In this assay, the NTCP prey constructs were co-transformed with the bait control vector pTSU2-APP, from which the amyloid precursor protein APP is expressed. None of NTCP constructs showed any unspecific interaction with APP in this assay on SD-AHLW plates, whereas co-transformation was confirmed by growth on SD-LW plates (Figure 1D). In contrast, co-transformation of the yeasts with pTSU2-APP and the prey control vector pN_ub_G-Fe65 confirmed growth (positive interaction control) on the selective plates, whereas co-transformation of pTSU2-APP with the empty prey vector pPR3-N did not show any growth (negative interaction control) as expected (Figure 1E). Finally, the NTCP bait construct was co-transformed with the NTCP prey mutant constructs G_60_LXXXA_64_L (TMD2), G_233_LXXXG_237_L (TMD7), and G_60_LXXXA_64_L/G_233_LXXXG_237_L (TMD2/7), as well as with the wild-type NTCP prey construct for control (Figure 1F). The necessary amount of 3-aminotriazole (3-AT) to suppress unspecific interactions was previously determined to 25 mM (Noppes et al., 2019) and was also used here. In this assay, homodimerization of the NTCP wild-type bait and prey constructs was used as the positive interaction control and the amount of yeast cells counted for this interaction was set to 100% (Figure 1G). Interestingly, all mutant NTCP prey constructs revealed growth on the SD-AHLW plates after co-transformation with wild-type bait NTCP in several independent dripping experiments. However, counting of the absolute number of yeast colonies on the SD-AHLW plates then revealed clear differences from the wild-type control (Figure 1G). The total number of colonies was significantly lower for the G_60_LXXXA_64_L (TMD2) mutant and declined even more for the G_233_LXXXG_237_L (TMD7) mutant and the G_60_LXXXA_64_L/G_233_LXXXG_237_L (TMD2/7) double mutant. As in the yeast system it can not be differentiated if this drop of interaction results from less efficient intermolecular protein-protein interaction or from intramolecular changes affecting protein folding and sorting, this effect was analyzed in more detail in cell culture.

### Subcellular Localization and Sorting of Na^+^/Taurocholate Co-Transporting Polypeptide Mutants

**FIGURE 2:**
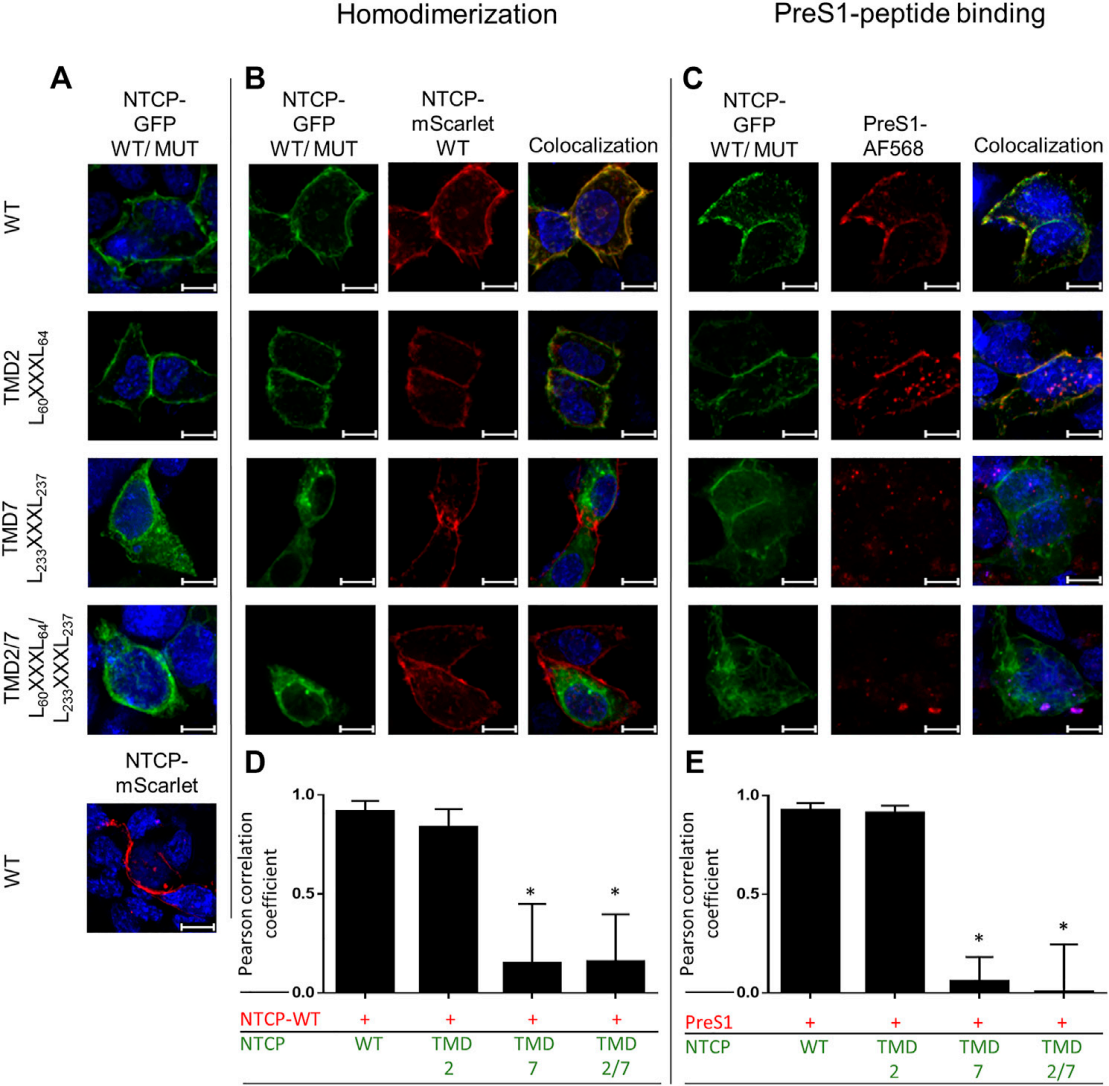
Colocalization of the wild-type and mutant NTCP constructs in HEK293 cells. HEK293 cells were seeded on coverslips and were **(A)** mono-transfected with the indicated wild-type or mutant NTCP-GFP constructs (green fluorescence) or were **(B)** co-transfected with wild-type NTCP-mScarlet (red fluorescence). After 48 h, slides were analyzed by fluorescence microscopy. Colocalization is indicated by fluorescence overlay (yellow). **(C)** Wild-type or mutant NTCP-GFP transfected HEK293 cells were incubated with 50 nM of the HBV/HDV-derived preS1-AF568 peptide for 20 min. After three washing steps with PBS, cells were subjected to fluorescence microscopy. Images represent maximum projections of z-stacks after deconvolution at 630 x magnification. Nuclei are indicated in blue, scale bars represent 10 µm. The Pearson correlation coefficient for the fluorescence overlay was determined **(D)** for NTCP homodimerization and **(E)** for preS1-AF568 peptide binding by measuring 10 representative individual cells with the Leica software LAS X. Data represent means ± SD. *Significantly different from control (NTCP-GFP(WT)-NTCP-mScarlet (WT) or NTCP-GFP(WT)-preS1-AF568) with *p* < 0.001 (one-way ANOVA with Dunnett’s multiple comparison test).

First, the question of subcellular localization and sorting of the NTCP mutants was addressed by transfections of HEK293 cells. For these experiments, NTCP-GFP and NTCP-mScarlet constructs served as templates for site-directed mutagenesis to enable expression of green fluorescent wild-type, G_60_LXXXA_64_L (TMD2), G_233_LXXXG_237_L (TMD7), and G_60_LXXXA_64_L/G_233_LXXXG_237_L (TMD2/7) NTCP-GFP fusion proteins. These constructs were either mono-transfected into HEK293 cells (Figure 2A) or were co-transfected with the red-fluorescent wild-type NTCP-mScarlet fusion protein (Figure 2B). The wild-type NTCP-GFP construct showed nearly complete colocalization with NTCP-mScarlet in the plasma membrane (Figure 2D). This also applied for the NTCP-G_60_LXXXA_64_L-GFP TMD2 mutant, which closely co-localized with NTCP-mScarlet and showed only slightly reduced Pearson correlation coefficient compared to wild-type NTCP-GFP (Figure 2D). In addition, plasma membrane expression of NTCP-GFP and NTCP-G_60_LXXXA_64_L-GFP was confirmed by binding experiments with the HBV/HDV-derived red fluorescent preS1-AF568 peptide (Figure 2C). As the preS1 peptide is unable to penetrate the plasma membrane, it only binds to NTCP molecules which are expressed at the plasma membrane. As indicated in Figure 2C, preS1-AF568 peptide binding clearly highlights membrane expression of wild-type and TMD2 mutant NTCP-GFP as well as close overlay between the green and red fluorescence signals (Figure 2E). In contrast, the G_233_LXXXG_237_L (TMD7), and G_60_LXXXA_64_L/G_233_LXXXG_237_L (TMD2/7) NTCP-GFP mutant proteins seemed to be miss-sorted and did not appear in the plasma membrane. Therefore, neither colocalization with the NTCP-mScarlet protein (Figures 2B,D), nor with the preS1-AF568 peptide (Figures 2C,E) could be detected. These experiments clearly show that mutation of the GXXXG motif in TMD7 leads to retention of the respective NTCP-GFP protein in intracellular compartments.

### Na^+^/Taurocholate Co-Transporting Polypeptide Homodimerization After Co-Immunoprecipitation

**FIGURE 3:**
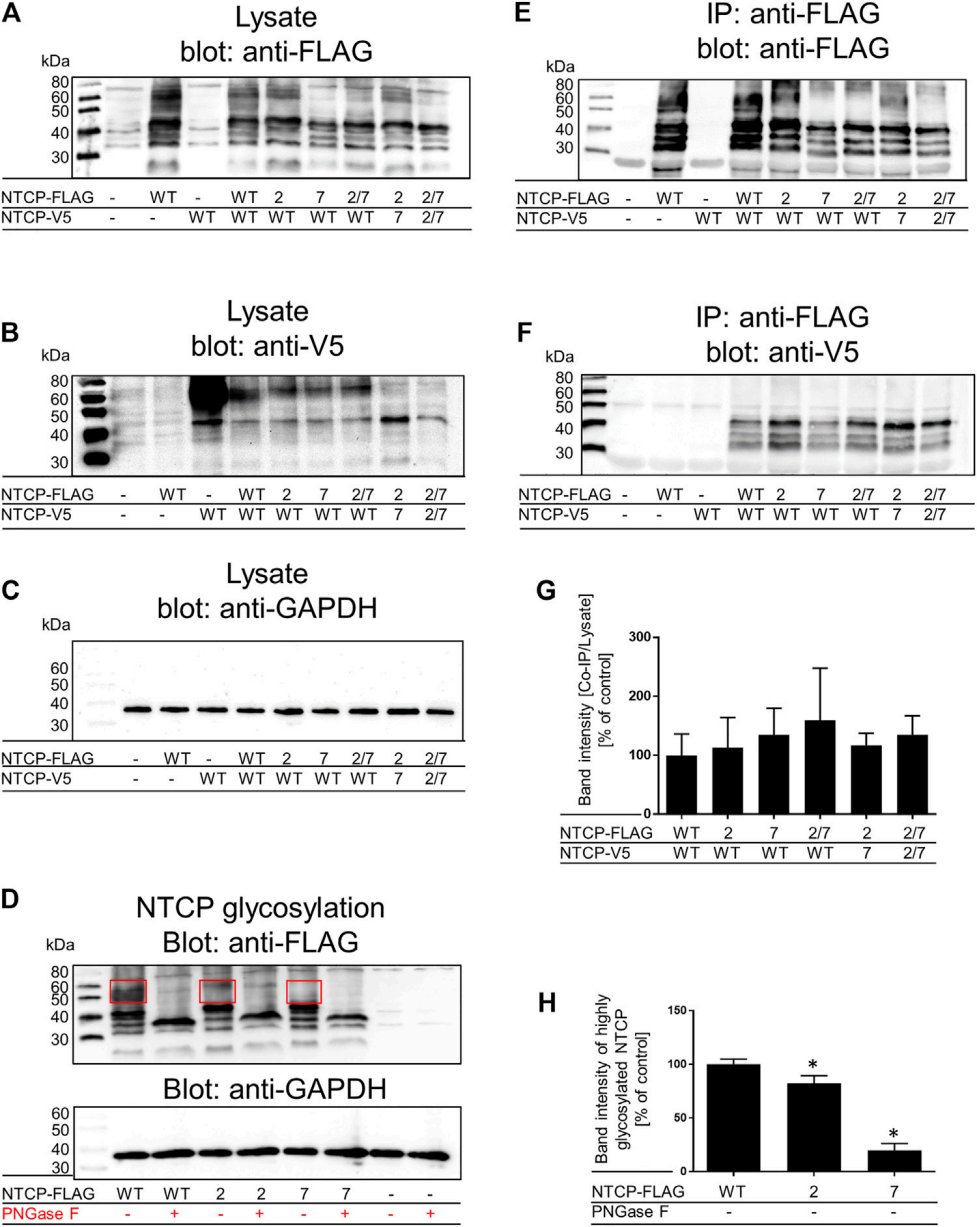
Co-IP and glycosylation status of wild-type and mutant NTCPs. Wild-type and mutant NTCP-V5-His and NTCP-FLAG constructs were mono-transfected or co-transfected into HEK293 cells as indicated. Cell lysates were analyzed by western blotting with **(A)** an anti-FLAG antibody, **(B)** an anti-V5 antibody or **(C)** and anti-GAPDH antibody. Cell lysates from un-transfected cells were used as negative control, mono-transfected cells served as positive controls. In the figure legend the NTCP TMD2, TMD7 and TMD2/7 mutants are briefly indicated as “2”, “7” and “2/7”, respectively. These cell lysates were also used for IP with anti-FLAG agarose and then were processed for western blotting with **(E)** an anti-FLAG antibody or **(F)** an anti-V5 antibody. **(G)** Band intensities of three independent co-IP experiments were measured using ImageJ and signal intensities from the IP anti-V5 signals were normalized for the lysate anti-FLAG signals. Ratios were related to the NTCP(WT)-NTCP(WT) interaction, which was set to 100%. Data represent means ± SD of three independent experiments, one of which is representatively shown under **(A–C)**, and **(E, F)**. **(D)** Cell lysates of wild-type and mutant NTCP-FLAG mono-transfected HEK293 cells were subjected to deglycosylation with PNGaseF. The glycosylated and deglycosylated samples were visualized by western blotting with an anti-FLAG antibody and an anti-GAPDH antibody. **(H)** Band intensities of the highly glycosylated wild-type and mutant NTCPs (bands with apparent molecular weight of 50–60 kDa as indicated by frame) were quantified using ImageJ. The values were then compared to wild-type NTCP-FLAG, which was set to 100%. Data represent means ± SD of three independent experiments. *Significantly different from wild-type with *p* < 0.05 (one-way ANOVA with Dunnett’s multiple comparison test).

Next, the question of intermolecular protein-protein interactions was analyzed by co-IP experiments that were performed after co-expression of a wild-type NTCP-V5-His protein together with FLAG-tagged G_60_LXXXA_64_L (TMD2), G_233_LXXXG_237_L (TMD7), or G_60_LXXXA_64_L/G_233_LXXXG_237_L (TMD2/7) NTCP mutants in HEK293 cells (Figure 3). As indicated by the NTCP controls (A, lysate anti-FLAG; B, lysate anti-V5), wild-type (WT) and mutant NTCP-FLAG and NTCP-V5 proteins were expressed at comparable amounts and band pattern after co-transfection (Figures 3A,B). In addition, the loading control (Figure 3C, anti-GAPDH) showed equal protein amounts for all samples. The multiple NTCP bands most likely represent different degrees of glycosylation of NTCP, ranging from the unglycosylated form (with an apparent molecular weight of ∼37 kDa), over less complex mannose glycosylated forms (at around 40 kDa), up to more complex glycosylated forms with molecular weights above 40 kDa (Figures 3A,B,D). After deglycosylation of the cell lysates with PNGase F, all these forms merged at a band with an apparent molecular weight of ∼37 kDa (Figure 3D), most likely representing the unglycosylated full-length NTCP. Smaller bands that still appear after PNGase F treatment possibly represent N-terminally truncated non-glycosylated forms of NTCP. Cell lysates of the double-transfected cells were subjected to IP with anti-FLAG agarose and the precipitates were analyzed by western blotting with anti-FLAG (IP, Figure 3E) or anti-V5 (co-IP, Figure 3F) antibodies. As indicated in Figure 3F, wild-type NTCP-FLAG as well as all NTCP-FLAG mutants co-precipitated the wild-type and mutant NTCP-V5 proteins to a certain degree. For quantification, the anti-V5 signals after co-IP were normalized for the anti-FLAG loading controls and data from three independent experiments are depicted in Figure 3G. No significant differences were observed between the NTCP wild-type and mutant co-IP signals. Of note, complex glycosylated forms of NTCP seemed not to be co-precipitated with NTCP-FLAG. At least no bands with apparent molecular weights above 40 kDa were detected with the anti-V5 antibody after co-IP (Figure 3F) in clear contrast to the loading control (Figure 3B). Appearance of these complex glycosylated forms of NTCP (apparent molecular weights of 50–60 kDa) were quantitatively analyzed for the wild-type NTCP as well as for TMD2 and TMD7 mutants. As shown in Figures 3D,H, under comparable protein amounts (loading control, anti-GAPDH) the TMD2 mutant showed slightly but significantly lower levels of complex glycosylated forms of NTCP, while in the TMD7 mutant these forms were almost completely absent.

### GXXXG/A Mutation and Bile Acid Transporter/Virus Receptor Function of Na^+^/Taurocholate Co-Transporting Polypeptide

**FIGURE 4:**
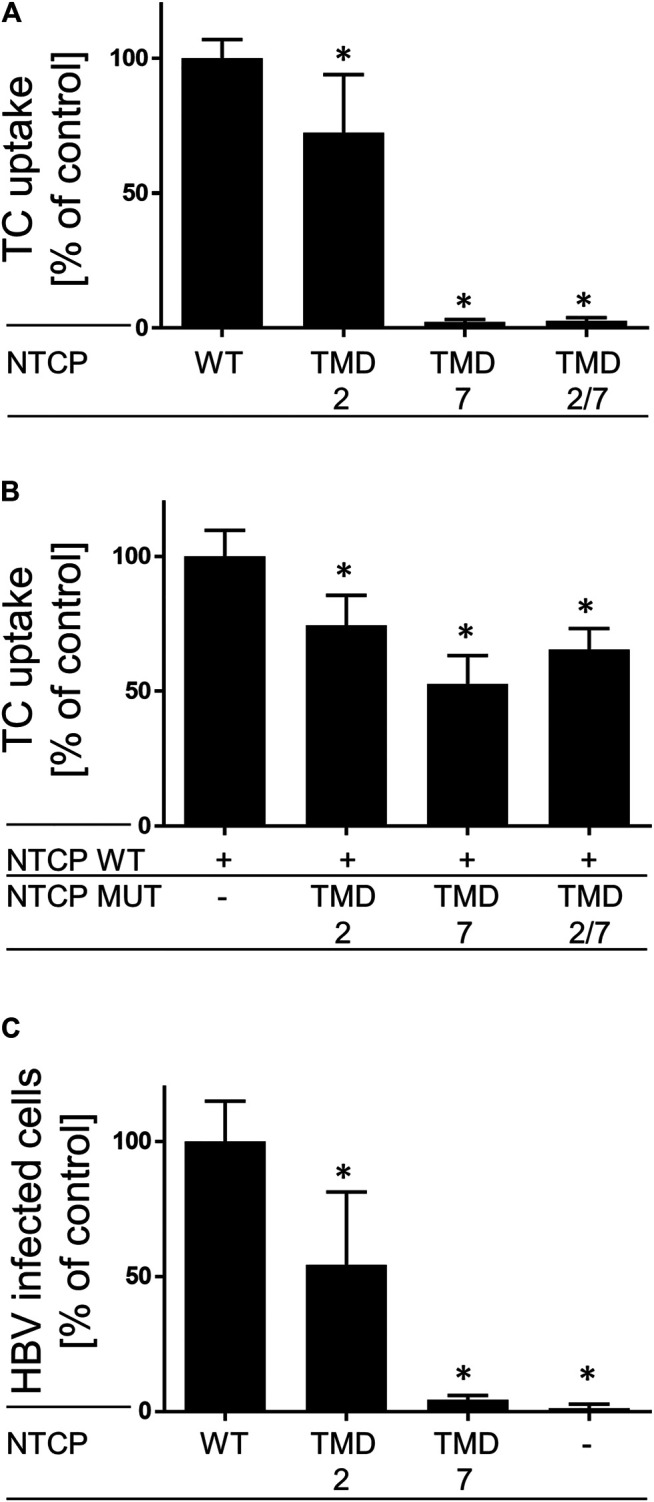
Bile acid transporter and HBV receptor functions of NTCP mutants. The indicated wild-type and mutant NTCP constructs were **(A)** mono- or **(B)** double-transfected into HEK293 cells as indicated. At 48 h post transfection, cells were incubated for 10 min with 10 µM [^3^H]TC and transport rates were determined compared to wild-type NTCP (set to 100%). Data represent means ± SD of combined data of two independently performed experiments with triplicate determinations. **(C)** HepG2 cells were transiently transfected with the wild-type and mutant NTCP constructs, respectively. Two days after transfection, cells were infected with HBV for 16 h. At 12 days post infection, cells were stained with an anti-HBc antibody. The numbers of HBV infected cells per well were counted using fluorescence microscopy. Data represents means ± SD of two independent experiments each with triplicate determinations (*n* = 6). *Significantly lower compared with wild-type NTCP with *p* < 0.05 (one-way ANOVA with Dunnett‘s multiple comparison test).

Finally, effects of GXXXG/A mutation on the bile acid transporter and virus receptor functions of NTCP were analyzed. To check if the physiological bile acid transport function of the NTCP mutants is still active, the respective constructs were transiently transfected into HEK293 cells for a subsequent measurement of taurocholate (TC) uptake. As shown in Figure 4A, the G_60_LXXXA_64_L (TMD2) mutant is still active in maintaining TC transport function at a level of about 75% compared to wild-type NTCP. In contrast, the TMD7 and TMD2/7 mutants, which do not reach the plasma membrane (see Figure 2B), were completely transport deficient (Figure 4A). Furthermore, it was investigated whether wild-type NTCP can be influenced in its transport behavior by co-expression and potential dimerization with one of these mutants. So, cells were co-transfected with wild-type NTCP and the respective mutant, and TC uptake was analyzed as before (Figure 4B). Co-expression of the G_60_LXXXA_64_L (TMD2) mutant with wild-type NTCP showed moderate reduction of TC uptake, similar to the mono-transfection condition (Figure 4A). In contrast, the G_233_LXXXG_237_L (TMD7) mutant maintained only about 50% of the transport rate of the wild-type NTCP (Figure 4B). Of note, formation of wild-type NTCP homodimers cannot be avoided here, so that transport rates are not expected to drop to zero in this experimental setup.

In order to analyze if mutation of the NTCP GXXXG/A motifs would have any effect on HBV susceptibility of the respective NTCP, wild-type and mutant NTCP constructs were transiently transfected into HepG2 cells and were used for *in vitro* HBV infection. At day 12 post infection, cells were subjected to fixation and the HBV core protein was stained using a polyclonal guinea pig-HBcAg antiserum (Figure 4C). After expression of the TMD2 mutant the *in vitro* HBV infection rate dropped to about 50% compared with wild-type NTCP. Even more pronounced, HepG2 cells expressing the TMD7 NTCP mutants were completely insensitive against HBV infection.

## Discussion

### Na^+^/Taurocholate Co-Transporting Polypeptide Dimerization and Hepatitis B Virus/Hepatitis D Virus Susceptibility

The aim of the present study was to analyze the molecular determinants and regulation of NTCP homodimerization. Homodimerization has been identified as a typical feature of NTCP (Bijsmans et al., 2012) and also other members of the SLC10 carrier family (Noppes et al., 2019), and more recently was also found to be relevant for the HBV/HDV virus receptor function of NTCP (Fukano et al., 2018). It was described that NTCP homodimerization is a prerequisite for cellular entry of the virus-NTCP complex and that the antidiabetic drug troglitazone is an appropriate inhibitor of this interaction. Since it was observed that the amount of preS1-peptide binding to NTCP dropped significantly when NTCP was kept in a monomeric state, this study claimed that blocking of NTCP homodimerization might be a novel strategy for HBV/HDV entry inhibition (Fukano et al., 2018). Furthermore, this study used NTCP peptide fragments, each displaying a length of 20 amino acids, which covered the whole NTCP sequence and investigated their role for NTCP dimerization and HBV/HDV susceptibility. The authors found that the peptide fragment derived from NTCP amino acids 221-240 prevented NTCP homodimerization and preS1-peptide binding. Interestingly, this peptide fragment covers the G_233_XXXG_237_ motif in TMD7 of NTCP that was closer analyzed in the present study. Therefore, it was hypothesized in the present study that this motif might be involved in NTCP homodimerization. In addition, a similar GXXXA motif in TMD2 was identified and analyzed. In order to investigate the role of these two sequence motifs of NTCP, different methods were applied that allowed investigation of NTCP sorting and homodimerization, as well as NTCP’s functions as bile acid transporter and virus receptor. We used the MYTH system, which allows detection of dynamic protein-protein interactions in living cells (Stagljar et al., 1998) and co-IP as a method more focused on static protein-protein interactions in cell lysates (Phizicky and Fields, 1995). Moreover, we checked the mutants for bile acid transport function as well as for susceptibility to HBV infection and preS1 peptide binding (Glebe et al., 2005). Finally, the sorting behavior of the mutated NTCP constructs was analyzed by fluorescence microscopy. As protein dimerization of membrane proteins is a complex process that involves correct folding and assembly in the ER, trafficking to the plasma membrane and dynamic conformational changes within the plasma membrane, this question cannot be addressed just by a single method. Furthermore, it has to be emphasized that all used methods direct quite different aspects of the dimerization and sorting process. This has to be considered when data seem not to be consistent between the different methods.

### The GXXXG Motif in Sorting and Dimerization

**FIGURE 5:**
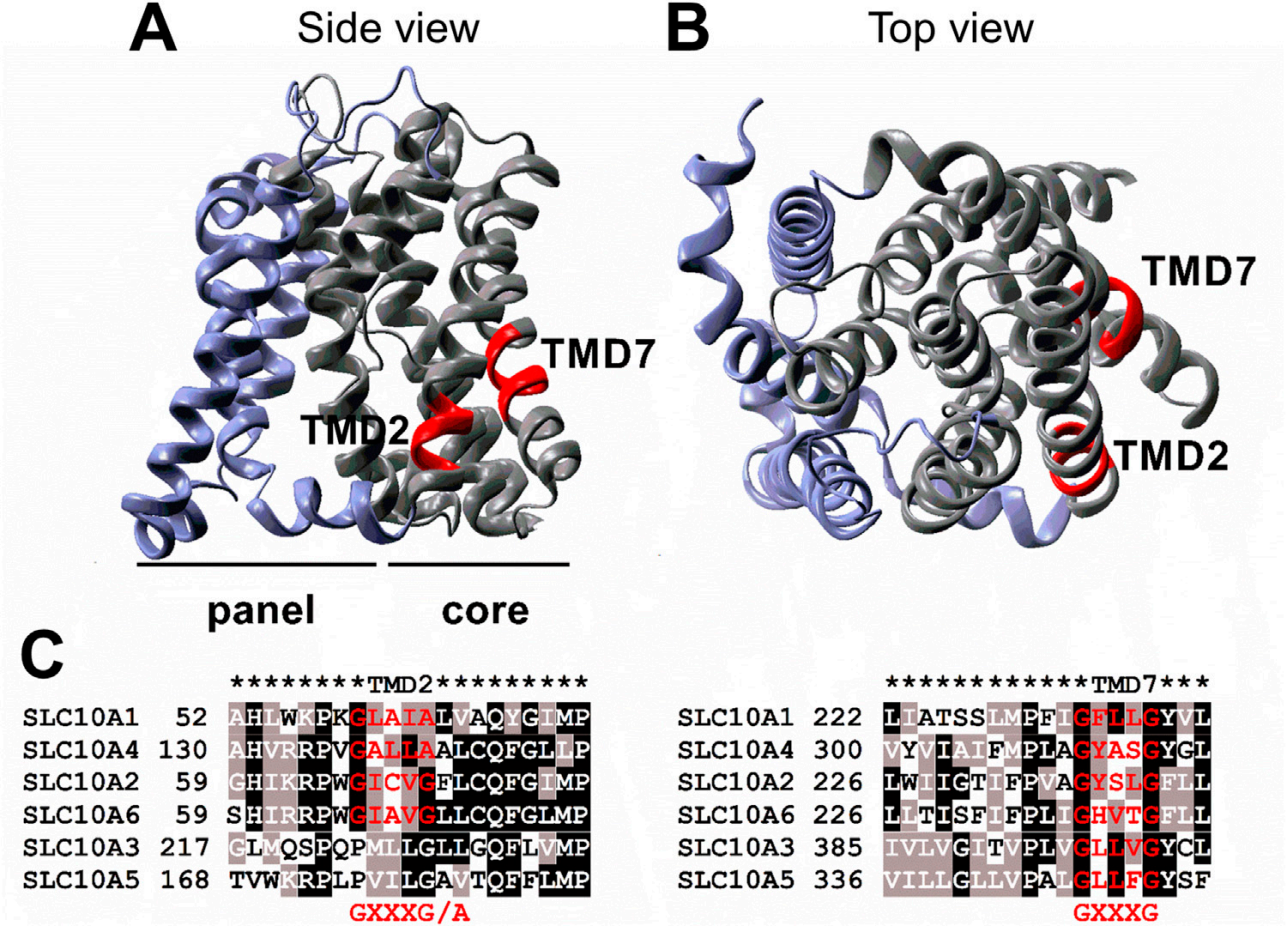
Location of the GXXXG/A motifs in TMD2 and TMD7 of NTCP. An NTCP homology model (**A:** side view; **B:** top view) was generated based on the crystal structure of a bacterial homolog by SWISS-MODEL as outlined under materials and methods. The panel domain is depicted in blue and the core domain is shown in grey. The GXXXG/A motifs in TMD2 and TMD7 are highlighted in red. **(C)** Sequence alignment of the protein sequences of the Solute Carrier Family 10 members A1-A6, with NTCP representing SLC10A1. Sequence alignment was performed with the EBI Clustal Omega alignment tool (www.ebi.ac.uk) using the protein sequences with the following GenBank Accession Numbers: SLC10A1/NTCP: NP_003040, SLC10A2/ASBT: NP_000443, SLC10A3: NP_062822, SLC10A4: NP_689892, SLC10A5: NP_001010893, and SLC10A6/SOAT: NP_932069. The alignment was visualized by BoxShade (https://embnet.vital-it.ch/software/BOX_form.html).

Since its discovery in human GpA as a dimerization motif in 1992 (Lemmon et al., 1992), the role of the GXXXG motif for homodimerization has been analyzed in a variety of transmembrane proteins (Teese and Langosch, 2015). This motif has a particular prevalence in transmembrane helices (Senes et al., 2000) and is often conserved among families of membrane transporters (Liu et al., 2002), as it is also the case for the solute carrier family SLC10. One GXXXG/A motif is present in TMD2 of the members SLC10A1, A2, A4 and A6, whereas the GXXXG motif in TMD7 is present in the SLC10 carriers A1-A6 (Figures 5A–C). In the present study, both sequence motifs were only analyzed for human NTCP (SLC10A1), but this might also be representative for the other SLC10 carrier family members. However, it has to be considered that the GXXXG/A motifs analyzed in the present study are not found in the bacterial ASBT sequence from *Neisseria meningitidis*, which was used as template for the NTCP homology model depicted in Figures 5A,B.

The MYTH experiments allowed the investigation of dynamic protein-protein interactions in living cells. Expression of all NTCP wild-type and mutant proteins was confirmed in the yeast cells, but their sorting could not be experimentally analyzed. Most interestingly, co-expression of the wild-type NTCP protein as a bait together with the TMD2 and TMD7 mutants of NTCP as preys revealed a significant decline in functional protein-protein interactions when one of these motifs, or both of them were mutated (Figure 1). However, in the MYTH system it cannot be differentiated whether intermolecular protein-protein interactions are directly affected or if the NTCP mutants are not properly folded and sorted, and as a secondary effect abolish functional protein-protein interaction. In order to differentiate between these possible mechanisms, additional experiments were performed in HEK293 and HepG2 cells that addressed sorting, membrane expression, protein-protein interaction and functionality of NTCP.

Fluorescence microscopy of respective GFP- or mScarlet-tagged wild-type and mutant NTCP proteins as well as binding experiments with the preS1-AF568 peptide then revealed that the TMD2 mutant was correctly sorted to the plasma membrane, where NTCP normally fulfills its role as a bile acid transporter and HBV/HDV virus receptor (Figure 2). Accordingly, the bile acid transport function and preS1 binding capability were almost completely preserved in this mutant. In contrast, mutation of the GXXXG motif in TMD7 (G_233_LXXXG_237_L) led to an intracellular accumulation of the NTCP mutant protein in the ER and Golgi compartments, accompanied by a loss of transport function (Figure 4A) and preS1 binding (Figures 2C,E). Based on this data, it can be supposed that the G_233_XXXG_237_ TMD7 sequence motif of NTCP is critical for correct protein folding or proper packing of the transmembrane segments that would be a prerequisite for its trafficking into the plasma membrane (Eilers et al., 2000). The incomplete glycosylation of the TMD7 mutant is an additional hint that protein folding and sorting are affected in this mutant, as only correctly folded proteins underwent complex glycosylation (Appelman et al., 2017). In contrast to these effects on protein folding and sorting, the TMD2 and TMD7 mutants showed no effect on co-IP together with the wild-type protein as indicated in Figures 3F,G. However, it is interesting to note that only apparently unglycosylated NTCP forms appear on the western blots after co-IP. This could mean that the co-IP experiments have limited validity for the complex glycosylated forms of NTCP that are expressed at the plasma membrane. The largely differing band pattern for the glycosylated forms of the NTCP-FLAG and NTCP-V5 proteins in these experiments most likely result from the different tags/plasmids used. Similar effects were also observed in previous studies (Bijsmans et al., 2012; Donkers et al., 2019). Although the MYTH experiments indicated effects of TMD2 and TMD7 mutation on direct protein-protein interaction of NTCP, this could not be confirmed in the co-IP experiments. This data indicates that the protein-protein interaction in the yeast might have been indirectly affected by misfolding and/or miss-sorting. Finally, the effects of TMD2 and TMD7 mutation on the susceptibility of NTCP to mediate *in vitro* HBV infection were of interest. Whereas, in HepG2 cells transiently transfected with the TMD7 mutant NTCP protein almost no infection was detected, the infection rate dropped to 50% for the TMD2 mutant (Figure 4). As this effect of TMD2 mutation was much higher than expected from the completely preserved preS1-binding capability, it can be speculated that the G_60_XXXA_64_ motif in TMD2 might be important for the conformational changes of the NTCP protein that occur after virus binding and that finally trigger the endocytosis of the virus-receptor complex. However, another explanation for this effect could be an uneven expression of wild-type and mutant NTCP after transient transfection of HepG2 cells. Anyhow, this effect needs further investigation in subsequent studies. The loss of HBV susceptibility for the TMD7 mutant can be explained by the localization of this protein in intracellular compartments (Figures 2A–C) making it impossible for the virus to bind to NTCP on the cell surface. Generally, it has to be considered that mutational studies on potential dimerization motifs only provide valuable and significant information, when the mutation does neither affect the folding and three-dimensional structure of the protein, nor its proper sorting. As both aspects seem to be affected in particular for the G_233_LXXXG_237_L TMD7 mutant, one only can conclude a role of this motif for folding and sorting of NTCP. However, a role of this motif for NTCP dimerization can not be completely excluded, as this would require experiments with a correctly folded and sorted protein, what can not be achieved by mutational analysis of NTCP. This is the major limitation of the present study.

### Role of the GXXXG Motif for Sorting, Dimerization and Transport Function in Other Carriers

The results from the present study go in line with previous studies that also analyzed the role of GXXXG motifs on the sorting and dimerization of membrane proteins. As an example, a study on the ATP-binding cassette transporter ABCG2 showed that GXXXG motifs promote proper packing of the transmembrane segments and this is important to form functional ABCG2 homodimers (Polgar et al., 2004). Respective glycine to leucine mutants lost this function, whereas glycine to alanine mutants were not affected (Polgar et al., 2004). This supports the hypothesis that the tight packing of transmembrane helices during dimerization is impaired when bulky residues like leucine are present. This might also apply for the G_233_LXXXG_237_L NTCP mutation of the present study by just disturbing proper TMD packing and thereby preventing the sorting process into the plasma membrane. Other studies with GXXXG mutation point to a direct role of this motif for protein-protein interaction. As an example, another G_144_XXXG_148_ motif of NTCP was analyzed in a previous study while searching for the interaction domain with the epidermal growth factor receptor (EGFR) that acts as an NTCP co-factor during internalization of the NTCP/virus receptor complex (Iwamoto et al., 2019). It was shown that G_144_AXXXG_148_A mutation strongly diminished the interaction between NTCP and EGFR, finally leading to decreased preS1-peptide binding and HBV/HDV infection rates in NTCP-transfected hepatoma cells (Iwamoto et al., 2019). Interestingly, the NTCP mutant G_60_LXXXA_64_L analyzed in the present study also showed a reduced susceptibility to *in vitro* HBV infection. However, in this case the signal of preS1-peptide did not differ from that of wild-type NTCP (Figure 2E). This data suggests that the G_60_XXXA_64_ motif in TMD2 is not involved in preS1-peptide binding, but more likely in the internalization step of the virus/receptor complex.

The role of the GXXXG motif was also analyzed for drug carriers of the SLC22 family, such as organic anion transporter 1 (OAT1) (Duan et al., 2011). The authors showed that mutation of the GXXXG motif in TMD2 of OAT1 led to a complete loss of membrane expression and transport activity. Similar findings were also obtained for individual SLC17 carriers and for the mitochondrial oxoglutarate carrier (Cappello et al., 2007; Courville et al., 2010; Ruprecht and Kunji, 2020). These findings reflect quite well the properties of the NTCP G_233_LXXXG_237_L mutant observed in the present study and clearly indicate the important role of GXXXG motifs for proper folding and sorting of membrane carriers.

## Conclusion

Prescreening of the TMD2 and TMD7 mutants of NTCP in the MYTH system revealed a clear drop in interactions after mutation of the respective GXXXG/A motifs. In the HEK293 cell model, membrane expression and bile acid transport activity were slightly reduced for the TMD2 mutant but were completely abolished for the TMD7 and the TMD2/7 mutants, while co-IP experiments still showed intact protein-protein interactions. Susceptibility for *in vitro* HBV infection in transfected HepG2 cells was reduced to 50% for the TMD2 mutant, while the TMD7 mutant was not susceptible for HBV infection at all. We conclude that the GXXXG/A motif in TMD2 and even more pronounced that in TMD7 are important for proper folding and sorting of the NTCP protein, and so indirectly affect glycosylation, homodimerization, and bile acid transport of NTCP, as well as its HBV/HDV receptor function. So, against our initial hypothesis, the GXXXG/A motifs in TMDs 2 and 7 of NTCP seem not to be the primary sites for direct NTCP homodimerization, but might be part of a larger interaction domain that needs to be elucidated.

## Data Availability

The original contributions presented in the study are included in the article/Supplementary Material, further inquiries can be directed to the corresponding author.

## Glossary

3-AT: 3-aminotriazole
ABCG2: ATP-binding cassette family G member 2
AF: Alexa Fluor
APP: amyloid precursor protein
ASBT: apical sodium-dependent bile acid transporter
BCA: bicinchoninic acid
C_Ub_: C-terminal part of ubiquitin
DMSO: dimethyl sulfoxide
DTT: dithiothreitol
EGF: epidermal growth factor
EGFR: epidermal growth factor receptor
GPA: glycophorin A
HBV: Hepatitis B Virus
HDV: Hepatitis D Virus
HGM: hepatocyte growth medium
HRP: horseradish peroxidase
IP: immunoprecipitation
LiOAc: lithium acetate
MUT: mutant
myr: myristoylated
MYTH: membrane-based yeast-two hybrid
NTCP: Na^+^/taurocholate co-transporting polypeptide
N_Ub_: N-terminal part of ubiquitin
OAT1: organic anion transporter 1
OD: optical density
PBS: phosphate-buffered saline
PEG: polyethylene glycol
SD: synthetically defined
SDS: sodium dodecyl sulfate
SLC: solute carrier
TBS: tris-buffered saline
TC: taurocholic acid
TMD: transmembrane domain
WT: wild-type
YPAD: yeast extract-peptone-adenine-dextrose
